# Evolution of a behavior-linked microsatellite-containing element in the 5' flanking region of the primate *AVPR1A *gene

**DOI:** 10.1186/1471-2148-8-180

**Published:** 2008-06-23

**Authors:** Zoe R Donaldson, Fyodor A Kondrashov, Andrea Putnam, Yaohui Bai, Tara L Stoinski, Elizabeth AD Hammock, Larry J Young

**Affiliations:** 1 Neuroscience Program, Emory University, Atlanta, USA; 2 Center for Behavioral Neuroscience, Emory University, Atlanta, USA; 3 Yerkes National Primate Research Center, Emory University, Atlanta, USA; 4 Division of Biological Sciences, University of California, San Diego, La Jolla, USA; 5 Zoo Atlanta, Atlanta, USA; 6 Vanderbilt Kennedy Center for Research on Human Development, Nashville, USA; 7 Department of Psychiatry and Behavioral Sciences, Emory University, Atlanta, USA

## Abstract

**Background:**

The arginine vasopressin V1a receptor (V1aR) modulates social cognition and behavior in a wide variety of species. Variation in a repetitive microsatellite element in the 5' flanking region of the V1aR gene (*AVPR1A*) in rodents has been associated with variation in brain V1aR expression and in social behavior. In humans, the 5' flanking region of *AVPR1A *contains a tandem duplication of two ~350 bp, microsatellite-containing elements located approximately 3.5 kb upstream of the transcription start site. The first block, referred to as DupA, contains a polymorphic (GT)_25 _microsatellite; the second block, DupB, has a complex (CT)_4_-(TT)-(CT)_8_-(GT)_24 _polymorphic motif, known as RS3. Polymorphisms in RS3 have been associated with variation in sociobehavioral traits in humans, including autism spectrum disorders. Thus, evolution of these regions may have contributed to variation in social behavior in primates. We examined the structure of these regions in six ape, six monkey, and one prosimian species.

**Results:**

Both tandem repeat blocks are present upstream of the *AVPR1A *coding region in five of the ape species we investigated, while monkeys have only one copy of this region. As in humans, the microsatellites within DupA and DupB are polymorphic in many primate species. Furthermore, both single (lacking DupB) and duplicated alleles (containing both DupA and DupB) are present in chimpanzee (*Pan troglodytes*) populations with allele frequencies of 0.795 and 0.205 for the single and duplicated alleles, respectively, based on the analysis of 47 wild-caught individuals. Finally, a phylogenetic reconstruction suggests two alternate evolutionary histories for this locus.

**Conclusion:**

There is no obvious relationship between the presence of the RS3 duplication and social organization in primates. However, polymorphisms identified in some species may be useful in future genetic association studies. In particular, the presence of both single and duplicated alleles in chimpanzees provides a unique opportunity to assess the functional role of this duplication in contributing to variation in social behavior in primates. While our initial studies show no signs of directional selection on this locus in chimps, pharmacological and genetic association studies support a potential role for this region in influencing V1aR expression and social behavior.

## Background

The neuropeptide, arginine vasopressin, acts centrally upon its V1a receptor subtype (V1aR) to modulate social behavior in a wide variety of species [1]. The remarkable degree of inter- and intra-species variation in the distribution of V1aR in the brain has been associated with variation in social behavior [2-5]. Because differences in central V1aR patterns of expression are likely due, at least in part, to differences in the regulation of the V1aR gene (*AVPR1A*), there has been considerable interest in identifying genetic candidate regions that may modulate V1aR expression in the brain. Such candidate regions may ultimately provide a genetic substrate for generating diversity in social behavior both across and within species.

Comparative studies in monogamous and non-monogamous vole species have suggested that variability in the 5' flanking region of the *AVPR1A *gene contributes to both variation in V1aR distribution patterns in the brain and in sociobehavioral traits. In particular, the composition of a microsatellite region located 626 base pairs (bp) upstream of the *AVPR1A *transcription start site (TSS) exhibits striking species differences in length and subtler individual length variation within species [3,5,6]. This inter- and intra-specific length variation is sufficient to drive differences in gene expression *in vitro *in a cell-type specific manner [3,7]. *In vivo*, individual variation in the length of this region in prairie voles is associated with differences in central V1aR patterns and variation in male-typical social behaviors [2,3].

These initial experiments in voles generated interest in the potential influence of variation in the human *AVPR1A *promoter on social behavior and central gene expression. A number of variable regions within the *AVPR1A *locus have subsequently been identified and used in gene association studies, including a microsatellite region termed RS3 located 3625 bp upstream of the human *AVPR1A *TSS [8-17]. RS3 is a complex repetitive region, unrelated to the vole microsatellite discussed above, composed of (CT)_4_-TT-(CT)_8_-(GT)_24 _where the combined number of CT and GT repeats varies from 16 to 50, yielding sixteen different alleles in the human population [8].

Preliminary evidence suggests that variation in this repeat element may influence *AVPR1A *gene expression in the brain. In post-mortem human hippocampus samples, longer RS3 repeat length has been associated with increased *AVPR1A *mRNA levels [16]. Several genetic association studies are also consistent with the hypothesis that variation in the RS3 element may contribute to variation in human sociobehavioral traits [10,11,13,15,16]. While only one study directly examines the link between RS3 variation and human social behavior [15], other studies, in whole or part, support an association between RS3 and traits that influence social behavior, including personality. For example, length variation in this region has been associated with altruistic behavior [16], and is also predictive of onset of first sexual intercourse in humans, a key reproductive behavior [10]. Additionally, within a study looking at the role of RS3 in creative dance performance, personality surveys indicate that RS3 is associated with individual scores on the Tellegen Absorption Scale and the Tridimensional Personality Questionnaire: Reward Dependence, which measure spirituality/empathy and social communication/need for social contact, respectively [13]. Beyond studies linking *AVPR1A *and various aspects of normal human behavior, there are now three independent studies linking this locus with autism, a disease hallmarked by deficits in social cognition [9,11,12]. Two of these studies have reported that specific alleles of RS3 are overtransmitted in autistic probands [9,12], and one of these studies suggests that variation in *AVPR1A *polymorphisms is predictive of the sociocognitive aspects of autism [11]. Taken together, these studies suggest that the *AVPR1A *locus, and in particular the RS3 region, may be important for determining variability in V1aR expression and social behavior.

**Figure 1:**
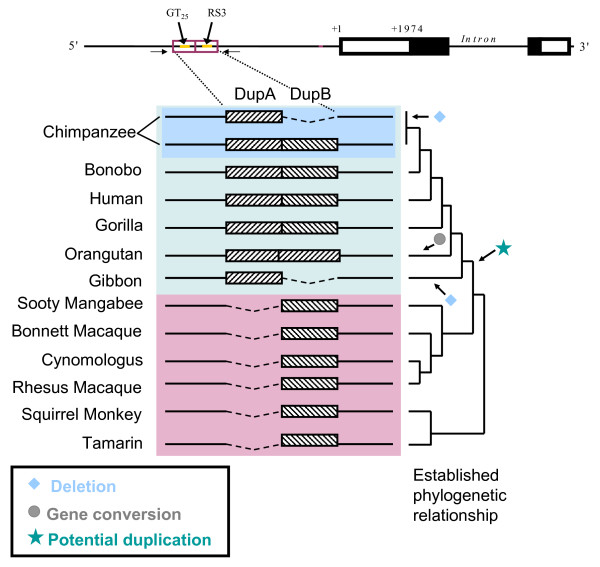
**Diagramatic representation of the DupA/B region in primates.** Accepted phylogenetic relationship is shown on the right [44,45]. Monkeys have a single copy of DupB that duplicated in the great ape ancestor. Gibbons and chimpanzees have alleles which have undergone a secondary loss of DupB. Orangutans have undergone a gene conversion event between DupA and DupB.

Within humans, the RS3 repeat region is housed within a larger, ~350 bp tandem duplicated region. The first of these duplicated regions, DupA, spans -3730 to -4074 bp and contains a GT_25 _microsatellite. The second block, DupB, spans -3382 to -3729 bp and contains microsatellite RS3 (Figure 1). In humans, DupA and DupB have ~87% sequence identity. Previously, we reported that while both humans and bonobos (*Pan paniscus*) carry both DupA and DupB, chimpanzees (*Pan troglodytes*) have only DupA, leading to a 357 bp difference between the chimpanzee and human *AVPR1A *upstream region [3]. This genetic difference in combination with behavioral differences among chimps, humans and bonobos, led us to further investigate the evolution of the DupA/B region as a potential candidate for determining differences in primate social behavior.

In this paper, we amplified and compared the *AVPR1A *RS3-containing 5' flanking region across a number of primate species, including a re-examination of the chimpanzee locus at a population level. We present two potential evolutionary histories for this region and describe a previously unidentified insertion-deletion (indel) of the Dup B region at this locus in chimpanzees. We also catalogue the microsatellite diversity for GT_25 _in DupA and RS3 in DupB in this region in five primate species and identify polymorphisms surrounding RS3 in two species of macaques. Finally, we perform initial studies to look for signatures of positive selection at this locus in chimpanzees.

## Results

### Primate AVPR1A evolution

#### Sequence analysis

We determined the sequence surrounding the RS3 *AVPR1A *upstream region in 13 primate species, which included 1 Strepsirrhini (norther greater galago [*Otolemur garnettii, Genbank: *EU760974), *2 Platyrrhini (squirrel monkey *[*Saimiri sciureus, Genbank: *EU760979] and golden lion tamarin [*Leontopithecus rosalia, Genbank: *EU7609780]), 4 Cercopithecoidea (sooty mangabey [*Cercocebus atys, Genbank: *EU760976], rhesus macaque [*Macaca mulatta, GenBank: *NW_001096629.1], cynomologus [*Macaca fascicularis, Genbank: *EU760982], and bonnet macaque [*Macaca radiata, Genbank: *EU760972]) and 6 Hominoidea (gibbon [*Hylobates lar, Genbank: *EU760981], orangutan [*Pongo pygmaeus, Genbank: *EU760977], gorilla [*Gorilla gorilla, Genbank: *EU760975], chimpanzee [*Pan troglodytes, Genbank: *EU780069 and EU78070], bonobo [*Pan paniscus, Genbank: *EU760973] and human [*Homo sapiens, Genbank: *NW_001838060]) species. We found that great apes contain a duplication of the microsatellite-containing region, while all six of the monkey species of both New and Old World origin have only DupB (see phylogenetic analysis for classification of this region; Figure 1). DupA and DupB are located in tandem with no intervening sequence between the two copies, and each of these duplicated regions is approximately 300 nucleotides, excluding the 30–80 variable nucleotide microsatellite region. The galago, the only prosimian species examined here, has a conserved region that corresponds with a portion of the DupA/B region but the sequences surrounding this area are highly diverged from the other primate species that we investigated, indicating that either this region is not fully represented prior to Simiiforms or we failed to identify a truly orthologous sequence. Additional investigation is needed to resolve the history of this region prior to Simiiformes.

In apes, the microsatellite contained within the duplicated region differs substantially between the two copies. The upstream DupA block houses a GT-only simple repeat while RS3 in all species that have DupB consists of a complex CT repeat immediately upstream of the GT repeat (Table 1). The single microsatellite in monkeys has both the CT and GT repeats, more similar to RS3 rather than to GT_25_. This is consistent with the hypothesis that DupB in apes is an exact ortholog of the single copy in monkeys with DupA being a more distant paralog. This hypothesis is also supported by sequence alignment, which consistently places the single repeat-containing block in monkeys with DupB in the apes.

**Table 1:** Microsatellite variation in the DupA/B region of six primate species

Species	N	GT_25 _(DupA)	RS3 (DupB)
Rhesus	5	not present	(CT)_2_CACTTT(CT)_11–15_(GT)_16–20_
Bonnet	4	not present	(CT)_2_CACTTT(CT)_11–19_(GT)_11–15_
Orangutan	5	T_1_(GT)_16–25_	(CT)_7_TT(CT)_5–10_(GT)_16–21_
Gorilla	9	T_1–3_(GT)_15–23_	(CT)_4_TT(CT)_7–13_(GT)_11–20_
Chimp long	2 long, 6 het	T_1–3_(GT)_20–26_	(CTTT)_2_(CT)_6–14_(GT)_8–24_
Chimp short	10 het, 13 short	T_3_(GT)_13–22_	not present
Human		T_3_(GT)_25_	(CT)_4_TT(CT)_8_(GT)_24_

Variability of the microsatellites within the DupA/B region is shown. Human variability is characterized in [10].

#### Phylogenetic analysis

**Figure 2:**
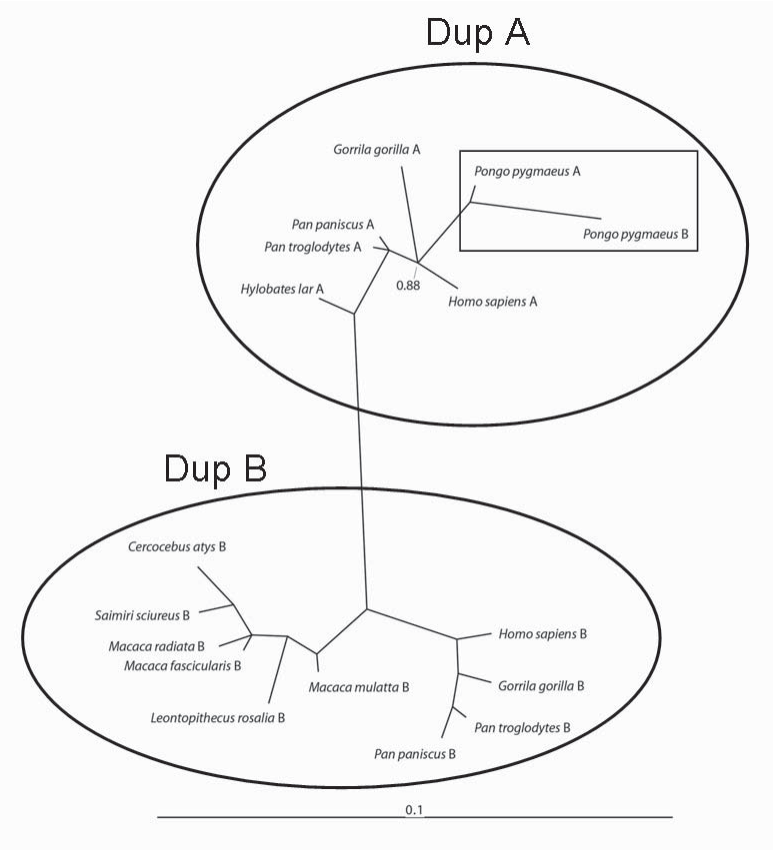
**Phylogenetic analysis of DupA and DupB in monkey and ape species.** DupA and DupB blocks for each species are indicated by "A" or "B," respectively, following the species name. With the exception of the orangutan (indicated by rectangle), all apes showed separate clades for DupA and DupB (indicated by circles). This provides strong support for an orthologous relationship between DupB and the monkey microsatellite-containing region. Clustered phylogenetic placement of the orangutan duplicated regions is consistent with a gene conversion event occurring between the two copies.

To test the hypothesis suggested by the sequence analysis that the monkey repeat-containing block is more closely related to the DupB region in apes, we reconstructed the phylogeny of the DupA/B region. To achieve this, we used a multiple alignment of all copies of the duplicated blocks excluding both of the microsatellite regions. With the exception of the orangutan, all apes showed separate clades for DupA and DupB, providing strong support for an orthologous relationship between the single monkey region and the DupB copy of this region in apes (Figure 2). The orangutan DupB region is more similar to its own DupA region than the DupB region of other apes. This observation, coupled with the phylogenetic placement of orangutan DupA and DupB regions, strongly supports a gene conversion event between the two copies, which was observed in all alleles in the five individuals we investigated. Gene conversion is thought to occur as a result of misalignment of the Holliday complex during recombination and results in the apparent replacement of one genetic region with another. Within orangutans, gene conversion events appear to have converted portions of DupB to DupA at some point in the past. Interestingly, the conversion events do not appear to include the microsatellites of the duplicated regions in orangutans, with the orangutan DupA region containing a GT repeat and the DupB region containing both the CT and GT repeats (Table 1), implying more than one short conversion event spanning the duplicated region in the orangutan.

There are two possible evolutionary explanations for the phylogenetic clustering of the ape DupB region with the single copy of this region found in monkey species (Figure 2). Either there has been an increase in the rate of evolution of the DupA region in apes relative to DupB, or multiple losses of the DupA region in the common ancestors of the monkeys examined in this study. The former explanation is more parsimonious, since the latter hypothesis requires at least two independent losses of the DupA region, in the common ancestors of both Old and New World monkeys. Although we sequenced this region in the prosimian species, the northern greater galago, the region was too far diverged to resolve these two alternative hypotheses. Therefore, more sequence data from other outgroup species are needed to resolve the evolutionary history of this region with a higher degree of confidence.

#### Microsatellite variation

Both the GT_25 _and RS3 microsatellites were variable across individual alleles in all of the primate species we investigated (5 rhesus macaques, 4 bonnet macaques, 5 orangutans, 9 gorillas, and 25 wild born chimps; Table 1). Because chimpanzees have two alleles that differ for the presence of DupB (see below for additional information), we performed separate analyses of these sequences for long and short alleles (genotypes, n = 2 long/long, 10 long/short, and 13 short/short individuals). Both macaque species have only the DupB region and show similar levels of RS3 microsatellite variability at this locus with slightly longer repeats occurring more commonly in rhesus. Among great apes similar levels of variability were seen for both DupA and DupB microsatellites. We did not find a marked difference in the range of length variability in the species we investigated and that previously reported for humans [10]. However, because our sample size was relatively small, it is possible that we missed low frequency alleles and a more thorough analysis should be undertaken if these microsatellites are used in association studies in the future.

#### Macaque sequence diversity

**Figure 3:**
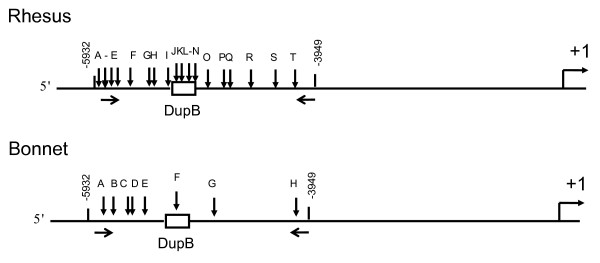
**Schematic representation of mutations we identified in macaques surrounding DupB**. The polymorphisms are described in Tables 2 and 3.

Because both rhesus and, to a lesser extent, bonnet macaques are used as animal models in many areas of research, including behavior, we undertook a study to catalogue the sequence diversity surrounding RS3. An assessment of the variability in this region provides a tool for future genetic association studies of this locus in these organisms. We sequenced ~2 kb of sequence surrounding RS3 in both rhesus and bonnet macaques and identified a number of polymorphisms (Figure 3, Table 2 and 3). Although these animals were captive born, every effort was made to obtain a diverse sample, and none of the individuals we investigated shared a first degree relative. Among 5 rhesus macaques of Indian origin, we identified 14 SNPs, and 5 indels. In contrast, in the same region in 4 bonnet macaques, we identified only 6 SNPs and 1 indel. Along with variation in RS3, these polymorphisms can be used in future genetic association studies. However, as a note of caution, rhesus macaques have a 5 bp indel 76 bp upstream of RS3 and care should be taken when designing potential primers for amplification of RS3 so that this indel does not influence the interpretation of microsatellite length.

**Table 2:** Variation in the rhesus *AVPR1A *RS3 and surrounding region

ID	Position	Polymorphism	Composition
A	2068771	SNP	g/t
B	2068763	SNP	a/t
C	2068762	SNP	a/t
D	2068759	SNP	c/a
E	2068710	INDEL	t/-
F	2068644	SNP	c/t
G	2068416	SNP	g/t
H	2068408	INDEL	c/-
I	2068266	INDEL	gttt/-
J	2068077	INDEL	gagta/-
K	2068001	STR (RS3)	(ct)_11–15_(gt)_16–20_
L	2067858	SNP	t/c
M	2067816	SNP	t/c
N	2067810	SNP	t/c
O	2067488	SNP	t/c
P	2067407	SNP	g/a
Q	2067392	SNP	a/t
R	2067351	INDEL	unresolved
S	2067188	SNP	g/a
T	2067056	SNP	t/c

Polymorphisms were identified in the ~2 kb surrounding RS3 in 5 rhesus macaques. Sequence location was derived from GenBank: NW_001096629.1.

**Table 3:** Variation in the bonnet macaque *AVPR1A *RS3 and surrounding region

ID	Position	Polymorphism	Composition
A	97	SNP	t/a
B	149	SNP	t/a
C	393	SNP	g/a
D	396	SNP	g/a
E	555	INDEL	a/a
F	857	STR (RS3)	(ct)_11–19_(gt)_11–15_
G	1216	SNP	t/c
H	1817	SNP	g/c

Polymorphisms were identified in the ~2 kb surrounding RS3 in 4 bonnet macaques. Base pair position indicates location within Genbank: EU760972.

#### Chimpanzee sequence diversity

**Figure 4:**
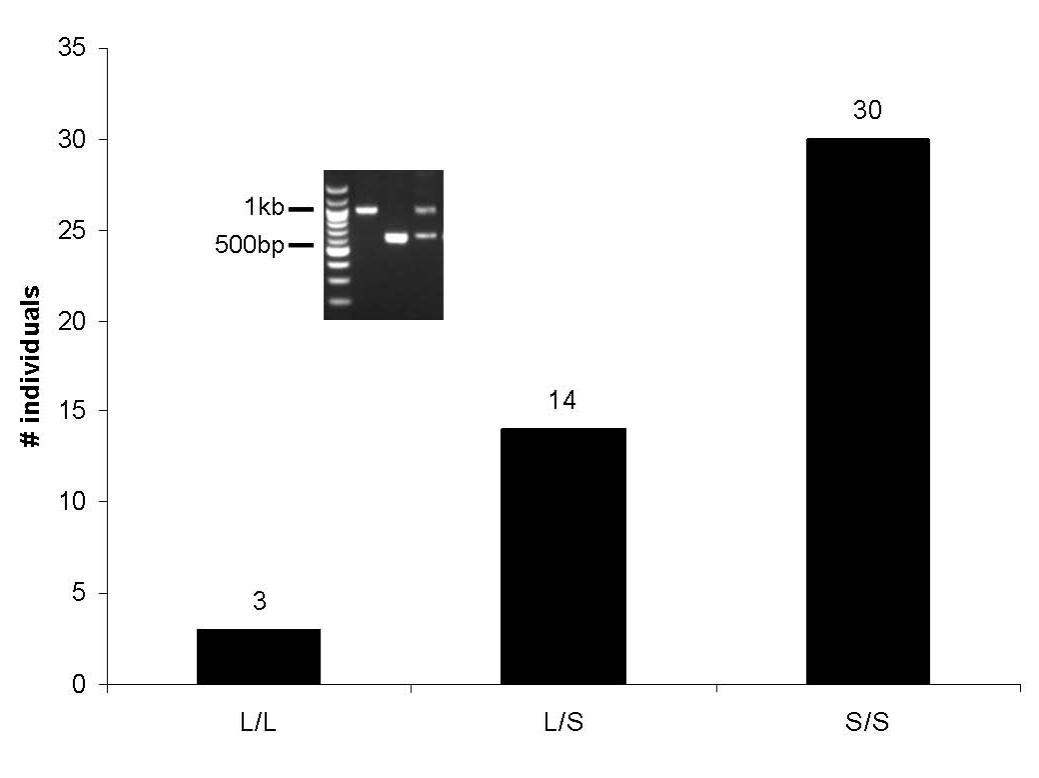
**Wild chimpanzees are polymorphic for the presence of DupB with short alleles having a prevalence of 0.795 and long alleles 0.205**. The graph shows the distribution of genotypes in wild-born chimps of primarily West African origin.

Inconsistent data for the DupA/B region obtained from the current version of the chimp genome (GenBank: NW_001223153) prompted us to obtain an independent sequence for this region. We found that chimpanzees are polymorphic for the presence of the DupB region, resulting in two alleles that vary by ~350 bp. In order to determine whether this polymorphism, originally identified in captive chimps, was observed in wild chimp populations, we genotyped 43 wild-born chimpanzees of primarily West African origin (35 from M.D. Anderson Cancer Center, 8 from Yerkes National Primate Center). Within this sample the frequency of the short allele (DupA only) is 0.795 and the long allele (DupA and B) is 0.205. The genotype frequencies are shown in Figure 4 and appear to be in Hardy-Weinberg equilibrium (χ^2 ^= 0.567, df = 2, p = 0.753).

#### Tests of neutrality at the chimp *AVPR1A *locus

**Figure 5:**
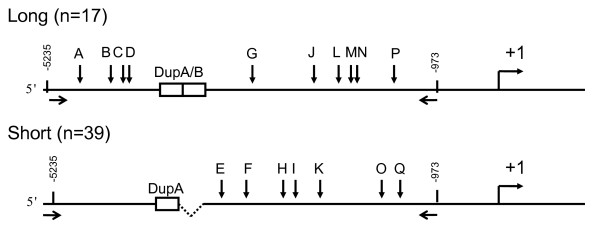
**Schematic representation of mutations we identified in the chimp *AVPR1A *upstream region.** Allelic frequencies for each mutation are given in Table 4.

As the short and long alleles are in Hardy-Weinberg equilibrium, there is not evidence for directional selection for a particular allele. However, there may be positive selection for a particular polymorphism or haplotype within or linked to the locus. To investigate non-neutral evolution at this locus in wild chimpanzees we examined patterns of polymorphisms within the surrounding 4 kp region, including 1520 bp upstream and 2395 bp downstream of DupA/B. We focused on the areas surrounding the duplicated blocks rather than the duplicated region itself because of technical difficulties associated with sequencing a microsatellite-containing duplication. Evidence of hitch-hiking, as indicated by the spreading of polymorphism(s) within a population due to their linkage with a beneficial mutation, should be detectable within the surrounding regions we investigated. We sequenced alleles from 28 randomly selected wild-born chimps of primarily West African origin. There were 17 long and 39 short alleles present in the population. We identified 7 SNPs in the short allele and 9 SNPs and 1 single bp indel in the long allele (Figure 5, Table 4), and used this information to analyze neutrality at this region.

**Table 4:** Frequency of polymorphisms identified in the chimp *AVPR1A *upstream region

SNP	Position	Long allele (freq)	Short allele (freq)
A	4970134	**g/a (0.941)**	g/g
B	4970836	**t/c (0.881)**	t/t
C	4973029	**t/a (0.706)**	a/a
D	4972125	**t/c (0.765)**	t/t
E	4973183	g/g	**g/a (0.923)**
F	4973341	c/c	**c/t (0.949)**
G	4973370	**g/a (0.941)**	g/g
H	4973669	a/a	**a/c (0.769)**
I	4973746	c/c	**c/t (0.667)**
J	4973932	**c/- (0.882)**	c/c
K	4973952	g/g	**g/t (0.897)**
L	4974132	**t/g (0.706)**	g/g
M	4974257	**g/t (0.941)**	g/g
N	4974314	**a/g (0.941)**	a/a
O	4974775	t/t	**t/c (0.692)**
P	4974861	**c/t (0.941)**	c/c
Q	4974893	a/a	**a/c (0.897)**

Identified polymorphisms and their relative frequency are shown for alleles from 25 individuals. Base pair position indicates location within Genbank: NW_001223153.1. Frequency shown is for the first nucleotide listed for each SNP or indel and is calculated for either the long or short allele with respect to where the mutation is observed.

Levels of nucleotide diversity are 0.073% for *π *and 0.089% for *θ *[18]. These values are almost identical to previously reported average multilocus levels of nucleotide diversity in Western chimpanzees [19]. Diversity was also examined within the long and short alleles. In the long allele, *π *is 0.054% and 0.048% in the short allele. This reduction in diversity within allele classes compared to the pooled population occurs because all polymorphisms were exclusively linked to either the short or the long alleles, with no SNPs shared between the two haplotypes. Using the four gamete test [20] to identify regions of recombination within the locus revealed at least two crossover events. One of these crossover events is apparent within the short alleles and the other within the long alleles.

At the *AVPR1A *locus, Tajima's *D *and Fay and Wu's *H *did not deviate from neutral expectations [21,22]. Tajima's *D *is -0.86 (95% CI -1.52 to 1.83) and Fay and Wu's *H *is -1.63 (95% CI -3.5 to 1.46). Similar to these results, Fischer *et al. *[19]report an average multilocus Tajima's *D *of -0.23 in Western chimpanzees. There is a negative skew in Fay and Wu's *H*, which may suggest an excess of high-frequency-derived polymorphisms, however, this result is not statistically significant. Therefore, our analysis provides no evidence for non-neutral selection within this region in chimpanzee.

## Discussion

Although our data support two possible scenarios for the evolution of the *AVPR1A *DupA and DupB regions in primates, both of these scenarios require complex histories involving duplication, deletion, and gene conversion events (Fig 1). Similar to humans, the GT_25 _and RS3 microsatellites in DupA and DupB, respectively, are polymorphic in multiple primate species (Table 1). Chimpanzees exhibit a polymorphic loss of DupB, and while our initial studies did not reveal evidence of directional selection at this locus in chimps, further studies are needed to better understand the contribution of this region to brain V1aR expression and sociobehavioral traits (Fig 3).

While we did not undertake a rigorous sociobehavioral classification of the species we investigated, a brief assessment of the known social behavior of these species does not support a relationship between duplication within the DupA/B region and social organization or specific social traits, such as the ability to form pair bonds. For instance, humans and gibbons both form selective social bonds, known as pair bonds, but humans have both DupA and DupB while gibbons have only DupA (reviewed in [23,24]). Within macaques, where social organization has been well categorized, DupA/B architecture does not seem to co-vary with various aspects of social behavior. Using a scale developed by Thierry [25], rhesus and bonnet macaques fall into different grades of social organization and vary in nearly all of the 22 social traits assessed. However both of these species, like all the monkeys we investigated, have only the DupB region of the AVPR1A gene. However, this initial assessment is limited and work is needed to thoroughly characterize the relationship between the DupA/B region and sociobehavioral traits both across and within species. In particular, a common scale for assessing primate social organization, such as that already in use for macaques would greatly enhance cross-species comparisons.

Similar to our findings in primates, a recent study investigating the evolution of the vole *avpr1a *upstream repeat region in 21 species revealed that the presence or absence of this DNA element was not sufficient to predict social organization [26]. However, given the evidence suggesting that variation in this region is associated with gene expression and behavior in humans, it is possible that various duplication and deletion events and their effects on the flanking regions have influenced sociobehavioral traits during primate evolution.

Several lines of evidence support the hypothesis that evolutionary changes within the DupA/B region may have affected primate social behaviors via alteration of brain V1aR expression. For example, differences in similar repetitive regions within the *avpr1a *upstream region in voles influences *in vitro *gene expression and has been tied to *in vivo *variation in brain V1aR expression and in social cognition and behavior [3,7]. Taken together, phylogenetic data, along with molecular and gene association studies, suggest that while evolution may have derived multiple mechanisms for determining rodent social structure, variation in the *avpr1a *promoter is one means for generating diversity in social behaviors [27].

Human studies also provide provisional evidence in support of the hypothesis that variation in the RS3 region may mediate differences in brain V1aR expression and social cognition and behavior. V1aR is highly expressed within the human lateral septum, a brain region associated with social behavior in many species [28]. However, the distribution of V1aR differs strikingly between humans and rhesus macaque, mirroring differences we identified in the *AVPR1A *upstream region of these two species [29]. To date, several human association studies examining *AVPR1A *have been conducted, investigating two to four repeat polymorphisms within the *AVPR1A *5' flanking region and intron, one of which is RS3 [described in 8]. While gene association studies do not provide direct functional evidence, the association of RS3 with sociobehavioral traits, personality aspects, or autism in several studies suggests that RS3 is a promising genetic candidate region for influencing social cognition and behavior [9-13,15,16]. Additionally, an association between RS3 length and *AVPR1A *mRNA levels in the human hippocampus further supports a role for RS3 in potentially influencing gene regulation [16]. However, additional functional studies are needed to understand the specific influence of the DupA/B region on V1aR expression and social behavior in humans as well as other primate species.

In addition to investigating the evolutionary history for this locus, we also sought to characterize the diversity of the microsatellites in DupA (GT_25_) and DupB (RS3) within several species. Like in humans, these microsatellites have multiple length variants within rhesus and bonnet macaques, chimps, orangutans, and gorillas. Because of advances in non-invasive hair and fecal DNA collection, these polymorphisms can be genotyped in wild individuals. This is of particular importance because many great ape species are endangered [30,31], and we may be able to gain valuable insights into optimal conservation strategies for these organisms.

We also characterized 2 kb of sequence surrounding RS3 in both rhesus and bonnet macaque to identify the variability within these regions. Both of these organisms are commonly used in research and rhesus macaques have become a primary primate model for basic and applied biomedical research [32]. Interestingly, we found considerably more variation present in the rhesus than the bonnet macaque for this region. This is consistent with the findings of a study that looked at a polymorphism in the serotonin transporter upstream region, another gene that has been linked with complex behaviors. The authors of that study identified more variability in rhesus compared to other macaque species [33]. While the interpretation of this observation is difficult, our initial studies suggest that sufficient variability may exist at this locus in captive research populations to carry out high resolution association studies.

Because non-coding variation is known to mediate individual differences in gene expression and social behavior in prairie voles, the striking indel polymorphism we identified in chimpanzees is particularly interesting. Loss of the DupB region in this species resulted in a two alleles that differ by approximately 350 bp. This polymorphism occurs naturally in the African chimp population, where the short allele is approximately four times as prevalent as the long allele. Given that the ancestral state is the duplicated allele, we investigated the potential for selection to have acted upon this locus.

Nucleotide diversity for this region was similar to that reported previously for neutrally evolving regions in West African chimpanzees [19], and tests of directional selection were not significant. While this data initially suggests that the chimpanzee's *AVPR1A *promoter region is not under selection, there may be alternative explanations for our findings. The presence of population structure and metapopulation dynamics may bias the estimates of Tajima's *D *and Fay and Wu's *H *toward being more negative [22,34,35]. Consistent with this possibility, patterns of polymorphisms show that all 16 SNPs are polymorphic within either the short or long alleles but not in both. This observation would be expected if gene flow was occurring between chimpanzee populations with different frequencies of long and short alleles. Evidence of recombination within but not between long and short alleles supports this hypothesis, and it is known that western and central chimp populations have low levels of population structure when compared to each other [FST 0.29; 19]. Alternatively, lack of recombination between long and short alleles may also reflect a linked, polymorphic inversion of this region in chimpanzees. Future multilocus sequencing would reveal if the observed polymorphism patterns are resulting from gene flow or inverted alleles. Alternatively, the short allele may be in the process of being driven to high frequency or fixation. If chimps lost DupB very recently then the test for selection using polymorphism frequencies may not detect evidence of selection, especially if the region is in an area of low recombination.

Finally, however, these results should be approached with caution. The historical records do not pinpoint the exact origin of these chimps. The few complete records that exist indicate that the majority of these chimps came from West Africa, as is common for many chimps in captivity in the United States (pers. comm. Susan Lambeth, University of Texas M.D Anderson Cancer Center at Bastrop). Use of multiple samples of known origins would greatly help in elucidating the evolution of this locus in multiple wild chimpanzee populations. In addition, as the population of chimpanzees available for research in the US disappears due to a moratorium on chimp breeding [36], genetic studies enabled by our preliminary work will provide a means for addressing physiological and behavioral hypotheses in wild populations. Ultimately, though, to better fully assess the functional consequences of this polymorphism, transcriptional assays and behavioural association studies are needed.

*AVPR1A *remains an exciting candidate gene for mediating differences in the social behavior of many species and potentially contributing to diseases characterized by deficits in social cognition [1,37]. Specifically, RS3 and the surrounding duplicated region provide an opportunity to discover how variation in the primate *AVPR1A *upstream region may mediate differences in brain V1aR expression and social behavior.

## Conclusion

We report the sequence and evolutionary history of the microsatellite-containing DupA/B region in the 5' flanking region of the *AVPR1A *gene, which may have relevance for understanding the role of variation in brain *AVPR1A *expression as it relates to social cognition and behavior. This region has undergone duplication, deletion, and gene conversion events including polymorphic deletion of DupB in chimpanzees (Fig 1). Similar to humans, the microsatellites in this region are highly variable within multiple species (Table 1). While we did not find significant relationships between the presence or absence of this region and social organization or mating strategy, it is possible that the duplication and deletion of this region, or variation in length of the microsatellites within this region have influenced sociobehavioral traits during primate evolution. Our identification of polymorphisms in the *AVPR1A *upstream region in macaques and particularly the deletion of DupB in chimps provides an excellent opportunity for exploring the relationship between variation in this region and social cognition and behavior (Figures 3, 4, 5, Tables 2, 3, 4).

## Methods

### DNA extraction, amplification, and sequencing

Buccal, blood, or tissue samples were obtained from Yerkes Regional Primate Research Center (chimpanzee, n = 83; gibbon, n = 1; rhesus macaque, n = 1; sooty mangabey, n = 3; squirrel monkey, n = 1; bonobo, n = 2; and cynomologus, n = 4), Zoo Atlanta (gorilla, n = 14; orangutan, n = 6; and golden lion tamarin, n = 1), University of Texas M.D Anderson Cancer Center at Bastrop (chimpanzee, n = 35), Wake Forest University (bonnet macaque, n = 5; rhesus macaque, n = 4; and cynomologus, n = 4), and Duke Lemur Center (northern greater galago, n = 1). The human and bonobo sequences have previously been published [3] and were also verified here. Genomic DNA was purified using Gentra PUREGENE DNA purification kits (Minneapolis, MN).

For chimpanzee, bonobo, gorilla, orangutan, rhesus macaque, bonnet macaque, cynomologus, and sooty mangabey, we amplified a ~2kb region that included the human DupA/B element using the Epicentre Failsafe PCR System in premix E with forward primer 5'-GAGGATCACCTGAGCCTG and a reverse primer of 5'-GGCATAGTGCATGATAGTCC with an annealing temperature of 57°C for 30 cycles: 95°C, 5 min; 30×(95°C, 30 sec; 57°C, 30 sec; 72°C, 3.5 min); 72°C, 10 min; 4°C, hold. Because these primers were unable to amplify this region in squirrel monkey, tamarin, gibbon, or galago, we amplified these species with alternative primer sets. For squirrel monkeys, we used Epicentre Failsafe PCR System in premix B with forward primer 5'-ATCGATCTAGATATGCACTCATACATGTAAGC and a reverse primer of 5'-GAAGAGCTGAATTTGAGCAG with an annealing temperature of 58°C for 40 cycles: 95°C, 5 min; 40×(95°C, 30 sec; 58°C, 30 sec; 72°C, 1 min); 72°C, 10 min; 4°C, hold. For golden lion tamarin, we used Epicentre Failsafe PCR System in premix A supplemented with 1% DMSO with forward primer 5'-TTGTTGAGCATGGTAGCCTCT and a reverse primer of 5'-GAAGAGCTGCCTTTGAGCAG with an annealing temperature of 59°C for 40 cycles: 95°C, 5 min; 30×(95°C, 30 sec; 59°C, 30 sec; 72°C, 30 sec); 72°C, 10 min; 4°C, hold. For gibbon, we used the Epicentre Failsafe PCR System in premix E with forward primer 5'-ACCCTTCAAACAATGCAACC and a reverse primer of 5'-ATCATGCTTCCAAATACTGGC with an annealing temperature of 57°C for 30 cycles: 95°C, 5 min; 30×(95°C, 30 sec; 57°C, 30 sec; 72°C, 3.5 min); 72°C, 10 min; 4°C, hold. Although our region of interest did not appear to be present in the initial ENSEMBL 1.5× coverage release of the northern greater galago genome, we were able to identify regions with high homology to the human sequence on either side of the RS3 region. After generating primers within these regions, we were able to amplify ~4 kb of the galago *AVPR1A *upstream region using a long-range step down PCR with Epicentre Failsafe premix K with forward primer 5'-GCTGTGCATAGATACGCTGG and reverse primer 5'-CCATGGAATCGAAGAACATTTGC with an annealing temperature of 56°C, 55°C, 54°C for ten cycles each: 95°C, 5 min; 10×(95°C, 30 sec; 56°C, 30 sec; 72°C, 3.5 min); 10×(95°C, 30 sec; 55°C, 30 sec; 72°C, 3.5 min); 10×(95°C, 30 sec; 54°C, 30 sec; 72°C, 3.5 min); 72°C, 10 min; 4°C, hold.

In cases where multiple bands were present, all products were gel extracted separately and cloned into pCR 4-TOPO vector (Invitrogen, Carlsbad, CA) according to manufacturer's instructions. DNA from at least two positive colonies per gel-extracted product were purified using Qiaprep spin miniprep kit (Qiagen, Valencia, CA) and the region of interest was sequenced by Lark Technologies (Houston, TX) or MacrogenUSA (Rockville, MD). Sequences homologous to the human DupA/B region were verified, aligned, and edited manually as needed using VectorNTi (Invitrogen, Carlsbad, CA).

### Sequence and phylogenetic analysis

Sequences were aligned with the muscle program [38] and then the alignment was manually checked in the MEGA3 browser [39] for accuracy. The phylogeny was reconstructed using MrBayes [40] with 1 million iterations (mcmc ngen = 1,000,000 in MrBayes) with the General Time Reversible model (Figure 2).

### Microsatellite variation

In order to asses potential microsatellite variation, we sequenced both the GT_25 _repeat in DupA and the RS3 complex (CT)_4_TT(CT)_8_(GT)_24 _repeat in DupB where applicable in rhesus macaques (n = 5), bonnet macaques (n = 4), orangutans (n = 5), gorillas (n = 9), and wild born chimpanzees (n = 25). The DupA/B region was amplified and cloned using the methods described above. Two clones per individual were sequenced to determine microsatellite variation for these five species. For rhesus and bonnet macaques, which have only DupB and not DupA, we sequenced RS3 with primer 5'-AACTTAACCACAAGGCTGAGC. For orangutans, gorillas, and chimpanzees, we sequenced the GT_25 _repeat of DupA with primer 5'-GCATGGTAGCCTCTCTTTAAT and RS3 within DupB with 5'-CATACACATGGAAAGCACCTAA. Because chimpanzees are polymorphic for DupB, only 8 of the 31 individuals sequenced had RS3. While these analyses are not meant to be exhaustive, they are sufficient to verify the potential for using these microsatellites in future genetic association studies in various primates.

### Macaque sequence diversity

Using the previously described PCR conditions, we amplified 1983 bp surrounding the DupB region of rhesus (n = 5) and bonnet macaques (n = 4). This region corresponds to basepairs 2068920 to 2066938 in Genbank: NW_001096629.1 (rhesus macaque). PCR products were purified and cloned. Both purified PCR products and two clones per individual were sequenced with primers 5'-GAGGATCACCTGAGCCTG, 5'-GGCATAGTGCATGATAGTCC, 5'-TGAGTAGCTGCCTTTGAGC and 5'-CATGCTTGACTTGCAGCAC. SNPs and potential INDELs were identified visually from chromatograms of the sequenced PCR products using VectorNTi. Each potential SNP and insertion/deletion was verified and resolved using the sequences from the cloned PCR product.

### Determination of allele frequency in wild chimpanzees

We genotyped 47 wild-caught chimps of primarily West African origin from Yerkes Primate Research Center (n = 8) and University of Texas M.D. Anderson Cancer Center (n = 35) to determine the approximate distribution of long and short alleles in the natural chimp population. The DupA/B region was amplified from chimp genomic DNA derived from blood or buccal samples (see above for purification methods) using Epicentre premix I with forward primer 5'-GCATGGTAGCCTCTCTTTAAT and a reverse primer of 5'-CATACACATGGAAAGCACCTAA with an annealing temperature of 57°C for 30 cycles: 95°C, 5 min; 30×(95°C, 30 sec; 57°C, 30 sec; 72°C, 3 min); 72°C, 10 min; 4°C, hold). PCR products were resolved on a 1.8% agarose gel and genotype was determined visually (see Figure 3). These primers are located just outside the chimp DupA/B region and correspond with chimp/human nucleotide differences in order to decrease potential cross-species PCR contamination.

### Analysis of chimpanzee alleles for potential non-neutral evolution

#### Amplification and polymorphism identification

Upon discovery of both duplicated and non-duplicated alleles in natural chimpanzees, we undertook an experiment to identify evidence of non-neutral selection at this locus. We amplified a 4.2 kb region surrounding DupA/B ranging from -5235 bp to -973 bp (Figure 4) in 28 wild born chimps (genotypes = 3 long/long, 11 long/short, 14 short/short). The PCR reaction was carried out in Epicenter Failsafe PCR premix E, with forward primer 5'-GTTGTGCATACATATCCTGG and a reverse primer of 5'-CAGGTAATCAAAGAACATTTCC using a touch-down technique with the following conditions: 95°C, 5 min; 10×(95°C, 30 sec; 54.6°C, 30 sec; 72°C, 6 min); 10×(95°C, 30 sec; 53.6°C, 30 sec; 72°C, 6 min); 10×(95°C, 30 sec; 52.6°C, 30 sec; 72°C, 6 min); 72°C, 10 min; 4°C, hold). The PCR product was gel purified and the region surrounding the duplication but not the duplication itself was sequenced in both forward and reverse directions using primers A-N as outlined in Table 5. SNPs and potential INDELs were identified visually from chromatograms in VectorNTi. Each potential SNP and insertion/deletion was verified with at least two independent sequencing primers. The same PCR products were cloned into PCR-TOPO4 vectors according to manufacturer's instructions and multiple clones from each individual were sequenced with primers L-O of Table 5 in order to reconstruct individual alleles.

**Table 5:** Primers used to sequence chimp region of interest

Primer	Sequence	Position	Direction
A	5' GTGGTCAGGGTACAGCTTG	4974096	Fwd
B	5' TGTAAGGTGACAGATGGTGTGGCA	4970407	Fwd
C	5' TCCCACCCTCTCCTGGTGATTTAT	4974930	Rvs
D	5' ATGGGTGACAGAGTGAGACCTTGT	4970853	Fwd
E	5' CGGGCTTACATGTATGAGTGCAGA	4971532	Rvs
F	5' ATCCATCCACCTTGGCCTCTCAAA	4970636	Rvs
G	5' TGTGTATGGGAGGCATCAGGGTAT	4973109	Fwd
H	5' AAGCATGATCTGCATCTGTGCTGC	4973598	Fwd
I	5' TCCTGACTGAAATTGGCCAGAAGC	4974455	Rvs
J	5' GGAAATCCTGTAGGATCTGCACTGGT	4973948	Rvs
K	5' GGCTGAGCTTCTTCCTGGAACTTT	4973437	Rvs
L	5' CGTGGAATGTTTCTGTATAACGG	4974540	Fwd
M	5' TGCTGGCAACATTGAGACTACCTC	4971123	Rvs
N	5' TATGCAGAGATGCCTGACTG	4973484	Fwd
O	5' AGATTCACTGAGCCAGACTAAGGC	4974320	Rvs

Primers used to sequence the chimp *AVPR1A *upstream region to identify polymorphisms and resolve alleles. Position indicates location within Genbank: NW_001223153.1. Fwd = forward primer, Rvs = reverse primer.

#### Tests of neutrality

All silent sites were used excluding insertions and deletions. Levels of nucleotide variability were calculated using both Watterson's estimator, *θ*, [18] based on the number of segregating sites in the sample, and *π*, the average pairwise diversity per nucleotide [41].

To test the fit to the standard neutral model, two summaries of the distribution of polymorphism frequencies were used. Tajima's *D*[22] is a measure of the standardized difference between *π *and *θ*. Fay and Wu's *H *also measures the difference between *π *and *θ*, but weighs derived variants by the square of their frequencies [21]. The orthologous *Homo sapiens *sequence was used as an outgroup for Fay and Wu's *H*. Under the standard neutral model, both test statistics are expected to give values close to zero. The values of the observed Tajima's *D *and Fay and Wu's *H *were compared to neutral coalescent simulations with recombination using the program, *ms*[42]. 10^4 ^neutral simulations were performed using a point estimate of *θ *based on the observed data. A point estimate of 5.0 × 10^-4 ^for the recombination rate per base pair, *ρ*, was chosen from previous Western chimpanzee estimate [43].

## Authors' contributions

ZRD conceived the polymorphism identification studies, carried out molecular genetic studies and drafted the manuscript. AP and FAK conceived and analyzed the directional selection studies, performed phylogenetic analysis and sequence alignment, and helped to draft the manuscript. YB performed molecular genetic studies. TLS and EADH conceived the investigation of the evolutionary history of this region. LJY participated in study design and coordination and helped to draft the manuscript.
